# The Complete Genome Sequence of the Murine Pathobiont *Helicobacter typhlonius*

**DOI:** 10.3389/fmicb.2015.01549

**Published:** 2016-01-08

**Authors:** Jeroen Frank, Celia Dingemanse, Arnoud M. Schmitz, Rolf H. A. M. Vossen, Gert-Jan B. van Ommen, Johan T. den Dunnen, Els C. Robanus-Maandag, Seyed Yahya Anvar

**Affiliations:** ^1^Leiden Genome Technology Center, Leiden University Medical CenterLeiden, Netherlands; ^2^Department of Human Genetics, Leiden University Medical CenterLeiden, Netherlands; ^3^Department of Clinical Genetics, Leiden University Medical CenterLeiden, Netherlands

**Keywords:** *Helicobacter typhlonius*, genome assembly, single-molecule real-time sequencing, Pacific Biosciences, pathogenicity, methylation

## Abstract

**Background:** Immuno-compromised mice infected with *Helicobacter typhlonius* are used to model microbially inducted inflammatory bowel disease (IBD). The specific mechanism through which *H. typhlonius* induces and promotes IBD is not fully understood. Access to the genome sequence is essential to examine emergent properties of this organism, such as its pathogenicity. To this end, we present the complete genome sequence of *H. typhlonius* MIT 97-6810, obtained through single-molecule real-time sequencing.

**Results:** The genome was assembled into a single circularized contig measuring 1.92 Mbp with an average GC content of 38.8%. In total 2,117 protein-encoding genes and 43 RNA genes were identified. Numerous pathogenic features were found, including a putative pathogenicity island (PAIs) containing components of type IV secretion system, virulence-associated proteins and cag PAI protein. We compared the genome of *H. typhlonius* to those of the murine pathobiont *H. hepaticus* and human pathobiont *H. pylori. H. typhlonius* resembles *H. hepaticus* most with 1,594 (75.3%) of its genes being orthologous to genes in *H. hepaticus*. Determination of the global methylation state revealed eight distinct recognition motifs for adenine and cytosine methylation. *H. typhlonius* shares four of its recognition motifs with *H. pylori*.

**Conclusion:** The complete genome sequence of *H. typhlonius* MIT 97-6810 enabled us to identify many pathogenic features suggesting that *H. typhlonius* can act as a pathogen. Follow-up studies are necessary to evaluate the true nature of its pathogenic capabilities. We found many methylated sites and a plethora of restriction-modification systems. The genome, together with the methylome, will provide an essential resource for future studies investigating gene regulation, host interaction and pathogenicity of *H. typhlonius*. In turn, this work can contribute to unraveling the role of *Helicobacter* in enteric disease.

## Introduction

The genus *Helicobacter* has rapidly expanded since it was first proposed in Goodwin et al. (1989). Today, the genus includes 35 *Helicobacter* species (Euzeby, 1997), with several (putative) novel species having been discovered recently (Menard et al., 2014). Members of this genus are Gram-negative and are characterized by having highly motile, multiple sheathed flagella and a helical, curved or straight unbranched morphology (Goodwin et al., 1989). All known *Helicobacter* strains live in human and animal hosts, where they primarily colonize the gastrointestinal tract (Franklin et al., 2001). Infection with *Helicobacter* sp. has been shown to be endemic in many animal facilities worldwide (Fox et al., 1994; Zenner, 1999; Whary and Fox, 2004; Chichlowski et al., 2008). Although as pathobionts they are benign commensals in immune-competent animals, they can act as opportunistic pathogens in immune-compromised mice.

The *Helicobacter* genus is well known for its association with enteric-, gastric-, and hepatic disease. The extensively studied human pathobiont, *Helicobacter pylori*, has been proven capable of causing a persistent inflammatory response in the stomach resulting in a 10–20% lifetime risk of developing peptic ulcers and a 1–2% risk of developing gastric cancer (Graham, 1989; Marshall, 1993; Parsonnet, 1995; Fox et al., 1999). Pathology caused by rodent *Helicobacter* sp. is often similar to those seen in human enteric diseases, especially inflammatory bowel diseases (IBDs) (Franklin et al., 2001). Consequently, rodent *Helicobacter* sp. are frequently used to infect immune-compromised mice to study these conditions in more detail.

One species used for IBD modeling is *H. typhlonius.* This murine *Helicobacter*, characterized by its lack of urease activity, is a prevalent intestinal colonizer of laboratory and feral mice (Franklin et al., 2001; Parker et al., 2009; Lofgren et al., 2012; Wasimuddin et al., 2012). Infection with *H. typhlonius* can induce and promote the development of severe IBD and IBD-associated neoplasia in immune-compromised *Il10^−/−^* mice (Chichlowski et al., 2008). These characteristics make infection with this species very useful to study IBD pathogenesis and treatment (Franklin et al., 2001; Chichlowski et al., 2008). Recently, we have shown that *H. typhlonius* infection can also modulate non-colitis-associated intestinal tumor formation as tested in conditional *Apc* mutant mice (Dingemanse et al., 2015).

To further elucidate the role of *Helicobacter* in enteric-, gastric-, and hepatic disease, it is increasingly important to determine the genomic sequence of the strain under study. Extensive sequencing efforts have resulted in the complete genomic sequences for at least 9 *Helicobacter* species, including many different strains (EMBL European Bioinformatics Institute [EMBL-EBI], 2014), while 17 species have been partly sequenced (National Center for Biotechnology Information [NCBI], 2014). Access to the complete genome contributes to the identification of potential virulence factors, permits the investigation of tissue tropism and may help unveil the mechanisms of pathogenesis. In this study, we reveal the complete sequence of the *H. typhlonius* genome along with its global methylation state at single-nucleotide resolution.

The *H. typhlonius* MIT 97-6810 genome was sequenced using Pacific Biosciences single-molecule real-time (SMRT) sequencing technology. The resulting long, highly accurate reads were virtually free of context-specific biases (Eid et al., 2009), ensured uniform genome coverage and were capable of resolving large repeats and structural variations. Ensuing *de novo* assembly and annotation of the genome, we performed base modification and motif identification analysis. It has been shown that methylation is involved in maintaining genome integrity, gene regulation, host interaction, cellular defense and limiting transformation by destroying foreign DNA (Jeltsch, 2003; Wion and Casadesus, 2006; Gonzalez et al., 2014; Krebes et al., 2014; Roberts et al., 2015). Finally, we present our comparative genomic results on closely related murine pathobiont *H. hepaticus* (Franklin et al., 2001; Fox et al., 2011; Krebes et al., 2014) and human pathobiont *H. pylori*. In addition, the global methylation state of *H. typhlonius* is compared to those of *H. pylori* strains 26695 and J99-R3 (Krebes et al., 2014).

## Results and Discussion

### Genome Assembly and Annotation

**FIGURE 1:**
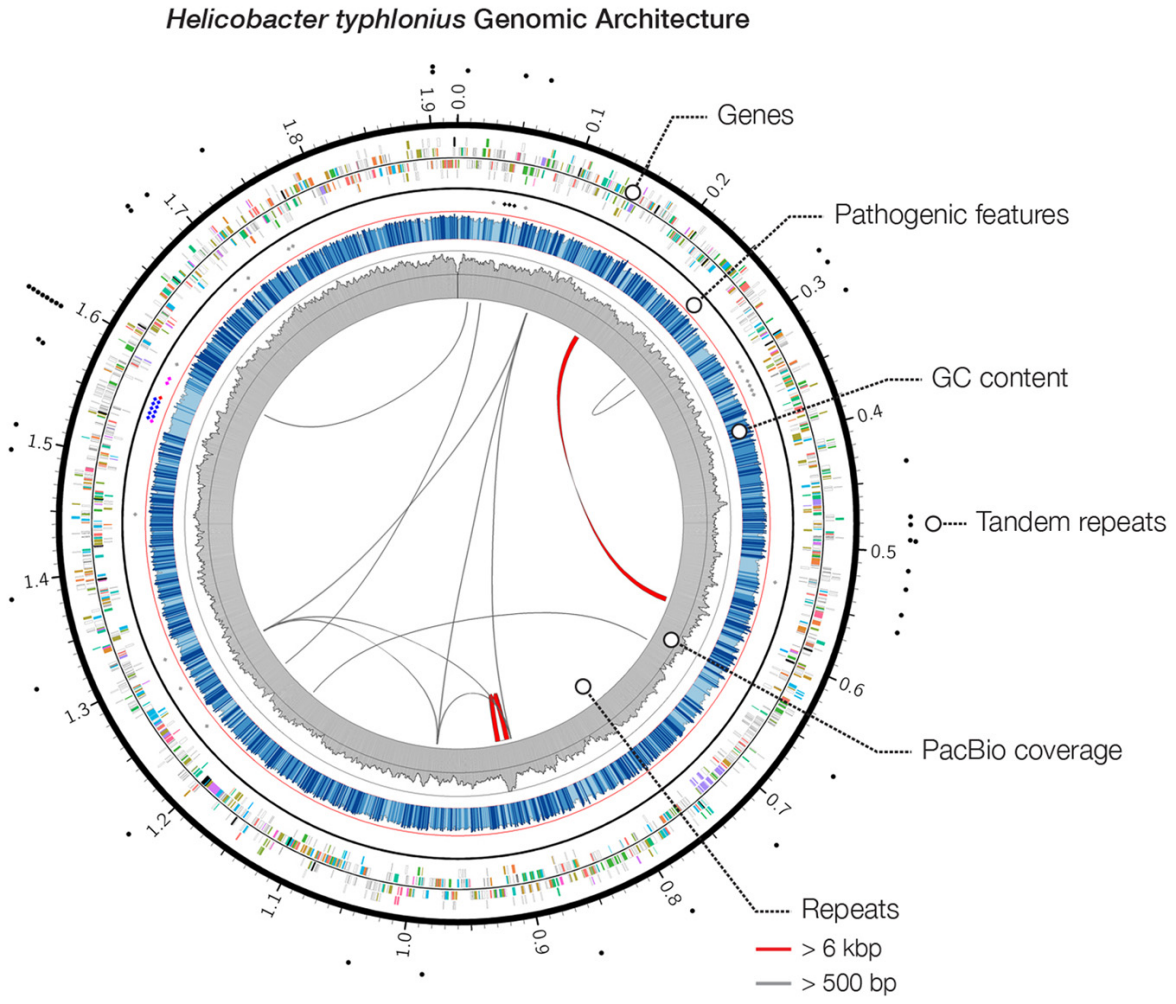
***Heliobacter typhlonius* genomic architecture.** Circos plot outlines the genomic features of *H. typhlonius*. On the outside, sites with short tandem repeats are indicated by black dots. The following ring (outside to inside) illustrates protein-encoding genes for the forward and reverse strand, colored by associated subsystem. Delineated separately (inner spots) are multiple pathogenic features, including cytolethal distending toxins (black), components of the T4SS (blue), virulence-associated protein 2 s (purple), a Cag pathogenicity island protein (red) and sequences that match pathogenic protein families in *H. hepaticus* (gray). The overall GC content is shown in blue, where light blue regions indicate <5% and dark blue >5% deviation from the average GC content (bin size: 1 Kb). The coverage profile of the SMRT sequencing reads is shown in gray (bin size: 1 Kb). On the inside, repetitive sequences and structural variations (>95% similarity at the nucleotide level) are shown throughout the genome with repeats >6 Kb colored red.

We performed SMRT sequencing to determine the complete genome sequence of *H. typhlonius* MIT 97-6810. In total 164,030 long (500- 29,940 bp), high-quality single-molecule sequencing reads were obtained (∼338 × coverage) (**Table 1**). Due to the nature of SMRT sequencing technology, long reads exhibit a relatively high randomly distributed error rate (Eid et al., 2009). Since most assemblers do not tolerate error rates greater than 5–10%, we used the hierarchical genome assembly process (HGAP) to correct sequencing errors. The resulting 4,157 corrected reads (**Table 1**) were assembled into a single 1,920,832 bp long contig with an average GC content of 38.8% (**Table 2**; **Figure 1**). To assess the accuracy and validity of the assembly, all sequencing reads were aligned to the assembled genome. The concordance between reads and reference sequence was found to be over 99.99% and no indication of sequence disagreement or coverage fluctuation could be found.

**Table 1:** Read statistics of 3 SMRT sequencing runs pre- and post-correction.

	PacBio RSII (Raw)	PacBio RSII (Corrected)^1^
Number of reads	164,030	4,157
Total nucleotides	649,035,578	37,634,528
Median read length	2,795 bp	9,053 bp
5th percentile	805 bp	686 bp
95th percentile	10,881 bp	16,281 bp
Maximum length	29,940 bp	20,234 bp
GC content	40.38%	38.86%
Coverage depth	337.89×	19.59×

*^1^Error-corrected PacBio reads generated by HGAP with seed length of 6,000 bp.*

**Table 2:** Single-molecule real-time (SMRT) *de novo* genome assembly statistics.

	SMRT *de novo*^1^
Number of reads	4,157
Sequencing depth	19.59×
Number of contigs	1
Bases in scaffolds	1,920,832 bp^∗^
GC content	38.8%
Accuracy	99.9890%

*^1^SMRT de novo assembly was carried out on corrected PacBio reads using Celera Assembler 8.1.*

*^∗^The total bases in the scaffolds were determined after circularization of the final assembly.*

The genome was examined for repeats and structural rearrangements. We found 13 long repeats and 42 short tandem repeats (STRs). There is one distinct region (genomic coordinates ∼885.2–907.9 Kb) that shows a complex repeat structure having relatively high coverage. This particular structure can also be seen in the assembly graph (Supplementary Figure S1). The repeat structure (size 22,672 bp) is slightly larger than the insert size of our sequence library (∼20 Kb), making it a challenging region to assemble. Therefore, although the sequencing reads seem to confirm the final genome sequence, we cannot exclude that the assembler could not fully resolve this region.

Next, the genome of *H. typhlonius* was automatically annotated using the RAST annotation service (Aziz et al., 2008; Overbeek et al., 2014). In total 2,117 protein-encoding genes (PEGs) and 43 RNA genes were identified, from which 890 PEGs (43%) were allocated to 278 annotated subsystems, biological processes or structural complexes realized by a set of functional roles (Overbeek et al., 2005) (**Table 3**). Subsequently, we estimated the location of the origin of replication (*oriC*) using Ori-Finder in conjunction with the DoriC database (Gao and Zhang, 2008; Gao et al., 2013). The genome was circularized accordingly with location of the predicted *oriC* at the start of the genome sequence (Supplementary Figure S2). The *dnaA* gene was found 11,655 bp upstream of the *oriC* region.

**Table 3:** Annotation statistics.

	*H. typhlonius*
Number of PEGs	2,117
Average PEG length	836 bp
Coding density	92.2%
PEGs assigned to subsystem	890 (42.0%)
Hypothetical proteins	747 (35.3%)
Number of rRNAs	4
Number of tRNAs	39

Recently, Sheh et al. (2014) deposited a draft-genome assembly of *H. typhlonius* MIT 98-6810 (also known as MIT 97-6810) in GenBank (ASM76576v1). They used the Illumina MiSeq platform to generate short reads that were assembled into 127 contigs which were subsequently scaffolded into 25 scaffolds. Compared to our assembly this assembly is fragmented and contains many scaffolding errors (Supplementary Figure S3). This fragmentation is likely caused by the nature of the Illumina data itself. Based on our assembly of the genome we could identify at least 13 repeated regions longer than 500 bp. Short Illumina reads (up to 300 bp) are unable to span such large repeats and structural variations, making it extremely difficult for the assembler to fully resolve these regions. Furthermore, DNA sequences having high or low GC content are notoriously difficult to PCR and therefore to sequence using second generation sequencing platforms. The PacBio RSII sequencer is not hampered by such characteristics; reads are long and there are virtually no context-specific biases. This enabled us to assemble the entire genome into a single continuous contig. Our assembly provides a comprehensive view of the genetic makeup and architecture of *H. typhlonius*.

### Pathogenicity

**FIGURE 2:**
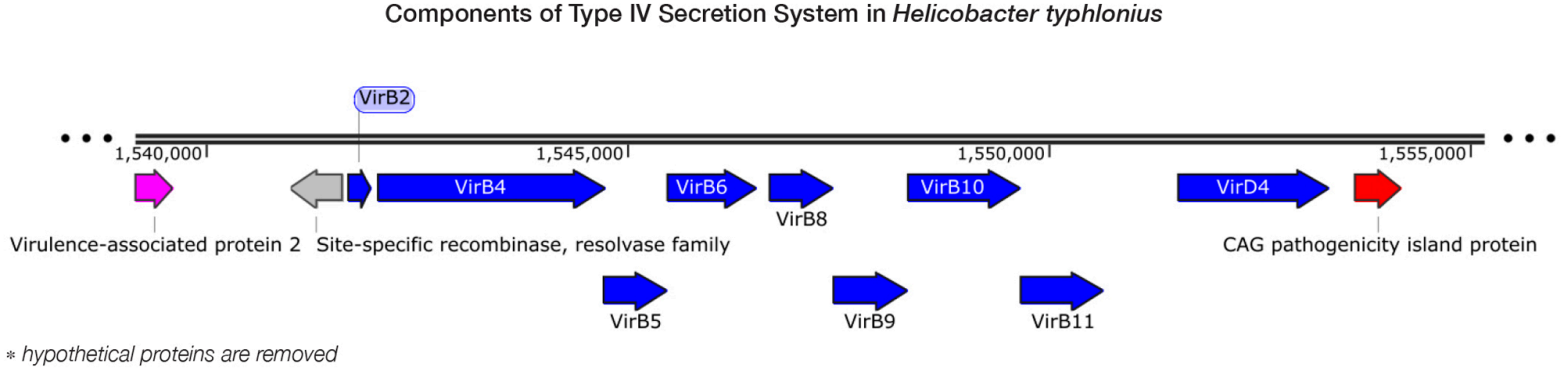
**Components of type IV secretion system in *H. typhlonius.*** Shown is a part of a putative pathogenicity island on the *H. typhlonius* genome (∼1.55–1.58 Mb). Components of the T4SS are colored blue. A virulence-associated protein and a Cag pathogenicity island protein are shown in purple and red, respectively. Hypothetical proteins have been omitted from this illustration.

Pathogenicity islands (PAIs) are distinct genetic elements that encode virulence-associated factors (Fox et al., 2011). They can often be detected by having a GC content, codon usage and *k*-mer frequencies, which are distinguishable from the rest of the genome owing to their origin through horizontal gene transfer (Che et al., 2014). The *H. typhlonius* genome contains one region with markedly lower GC content (∼34.2%) that is flanked by repeats at the 3′ end (∼1.53–1.60 Mb) (**Figure 1**). The size of this genomic island is estimated to be around 65.5 Kb and is located at 1,532,276–1,597,776 bp. This region contains 75 PEGs that constitute mostly hypothetical proteins (36 PEGs) but also includes many components of type IV secretion system (T4SS). The ability to secrete compounds including toxins is essential for virulence and survival (Fronzes et al., 2009). T4SS families can be divided into three classes based on functionality. First, T4SSs are involved in conjugation, a mechanism that enables the transfer of genetic material such as antibiotic resistance genes among bacteria (Dreiseikelmann, 1994). Second, T4SSs mediate DNA uptake from and release into their surroundings, further enabling genetic exchange (Hamilton and Dillard, 2006). Finally, T4SSs are directly involved in the transfer of protein effectors, including toxins, into eukaryote cells during infection (Fronzes et al., 2009; Terradot and Waksman, 2011). Each of these T4SS classes have been identified in *H. pylori* (Terradot and Waksman, 2011). T4SS typically consists of a collection of twelve proteins: VirB1–11 and VirD4. The presence of VirB2, VirB4-VirB6, VirB8-VirB11, and VirD4 in *H. typhlonius* was confirmed via RAST annotation (Supplementary Table S1). Additionally, using BLASTX, we observed strong evidence for the presence of VirB3 and VirB7 in *H. typhlonius*, whereas VirB1 was absent. Furthermore, cytotoxin-associated gene (*Cag*) PAI protein and 3 virulence-associated proteins were also present in the genome of *H. typhlonius* (**Figure 2**, Supplementary Table S1). The presence of a partial T4SS, a Cag protein and several virulence factors suggests that this region is a putative PAI.

Suerbaum et al. (2003) identified and characterized a PAI (HHGI1) in *H. hepaticus* ATCC 51449. This PAI spans 71 Kb and has a GC content of 33.2%. HHGI1 contains 70 open reading frames (ORFs) including pathogenic and virulent homologs, but they predominantly encode hypothetical proteins (Suerbaum et al., 2003). We could not confirm the presence of HHGl1 in *H. typhlonius*. Moreover, BLAST results of all 70 ORFs against the *H. typhlonius* genome retrieved very limited hits, except for one *H. hepaticus* gene, *HH0237*, that was partly found in *H. typhlonius. HH0237* is a homolog of a structural component of known bacterial type VI secretion systems (T6SS) (Fox et al., 2011).

We searched for genes encoding subunits of cytolethal distending toxins (CDTs), which are present in several Gram-negative pathogens, including *Helicobacters*. CdtA and CdtC subunits bind together to subsequently deliver an active subunit of the CdtB toxin (Fox et al., 2011). Each of these three subunits was found in the *H. typhlonius* genome (Supplementary Table S1). The active CdtB unit has been associated with a variety of biological functions including DNase I-like function, cell-cycle arrest, phosphatase activity, and apoptotic cell death (Ge et al., 2008). Loss of CDT functionality in CDT-deficient isogenic *H. hepaticus* mutants affects the capability to colonize the large intestine, resulting in milder symptoms of typhlocolitis upon infection in mice (Young et al., 2004; Ge et al., 2005; Pratt et al., 2006).

We used multiple tools to predict and identify additional putative virulence factors, antimicrobial resistance genes or pathogenic features. VirulenceFinder and ResFinder (Zankari et al., 2012; Joensen et al., 2014) did not detect any additional virulence- or antimicrobial resistance genes. PHAST (PHAge Search Tool) (Zhou et al., 2011) was used to detect and annotate prophage sequences, but none were found. PathogenFinder (Cosentino et al., 2013) reported 19 proteins that are linked to pathogenic protein families in *H. hepaticus*, comprising mostly hypothetical proteins (Supplementary Table S2).

### Comparative Genome Analysis

Phylogenetic analysis by Franklin et al. (2001) has demonstrated that *H. typhlonius* is closely related to *H. hepaticus*. This latter *Helicobacter* is a genuine murine pathobiont, capable of causing IBD, chronic hepatitis and liver cancer in numerous mouse models (Suerbaum et al., 2003; Fox et al., 2011). In turn, *H. hepaticus* is closely related to the human pathobiont and type species *H. pylori* (Franklin et al., 2001). The genome of *H. typhlonius* (1,92 Mbp) is somewhat larger than the genome of *H. hepaticus* ATCC 51449 (1.80 Mbp, accession NC_004917.1) and *H. pylori* 26695 (1.67 Mbp, accession NC_000915). The GC content is very similar for *H. typhlonius* (38.8% GC) and *H. hepaticus* (38.9% GC), while *H. pylori* (35.9% GC) deviates from the two having a considerably lower GC content.

**FIGURE 3:**
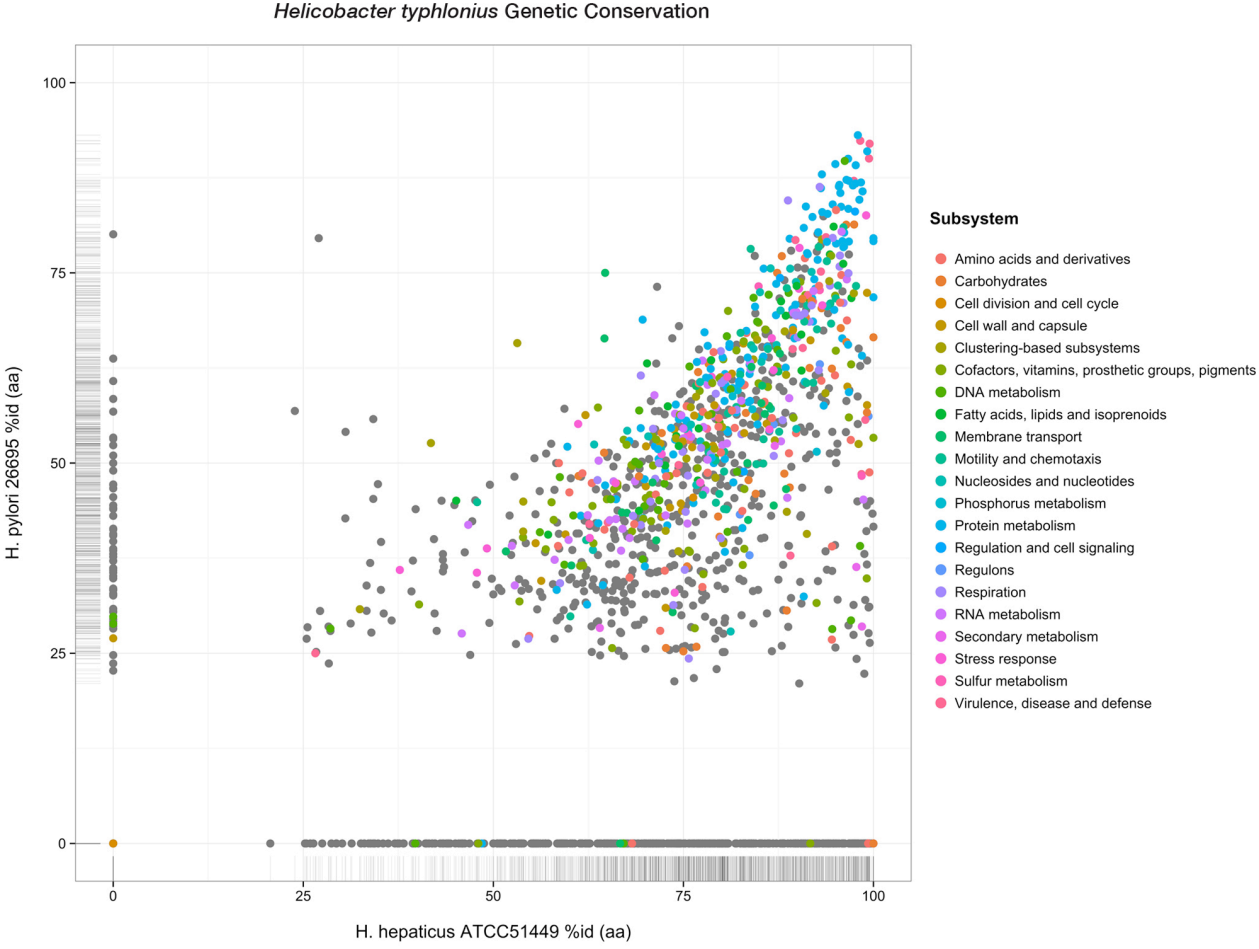
**Genetic conservation.** Scatterplot showing all protein-encoding genes of *H. typhlonius* and their respective orthologs in *H. hepaticus* and *H. pylori.* Similarity is expressed as the percentage of amino acid identity. Protein-coding genes are colored according to subsystem adopting the same color scheme as in **Figure 1.** PEGs colored gray do not have any association to a subsystem.

Although less PEGs are predicted for *H. hepaticus* ATCC 51449 and *H. pylori* 26695 (1,879 and 1,620 PEGs respectively), *H. typhlonius* sequence mostly resembles *H. hepaticus* as 1,594 (75.3%) of its genes were found as orthologous to genes in *H. hepaticus*. This number is significantly lower for *H. pylori* having only 1,170 (55.3%) orthologous genes. This is also evident from the amino acid identity of orthologs in *H. hepaticus* (76.4% AAI) compared to that of *H. pylori* (50.6% AAI) (**Figure 3**). The conservation of major subsystems in *H. hepaticus* and *H. pylori* varies, with specific subsystems being conserved higher in one over the other and vice versa (Supplementary Figure S4).

We also identified 468 PEGs that are unique to *H. typhlonius.* The great majority of these PEGs (386) constitute hypothetical proteins. There are nonetheless several annotated PEGs with predicted functions including two virulence-associated proteins, six glycosyl transferases, DNA recombination protein RmuC, DNA sulfur modification protein DndD, several PEGs that are part of restriction-modification (R-M) systems and two CRISPR-associated (Cas) proteins: Cas1 and Cas2. (Supplementary Table S3). Cas1 and Cas2 are part of a complete type II CRISPR-Cas system including Cas9 and a downstream CRISPR array containing 22 spacers that are located at 1,593,570–1,595,058 bp.

Furthermore, we compared the *H. typhlonius* genome against all other *Helicobacter* genomes available in The SEED genome database (Overbeek et al., 2005). A collection of 38 annotated PEGs with diverse functions was exclusively found in *H. typhlonius* (Supplementary Table S4). This set of PEGs determines the uniqueness of the *H. typhlonius* genome, representing 2.1% of this genome.

### Base Modifications and Associated Motifs

We have identified components of R-M systems in the *H. typhlonius* genome, some of which are present in *H. hepaticus* ATCC 51449 and *H. pylori* strain Shi470 as well (Supplementary Table S5). Many putative DNA methyltransferases (MTases) were found, indicating that it should be possible to detect different types of methylation. Of the 18 DNA MTases, 9 orthologs were also present in *H. hepaticus* ATCC 51449 (average 84.2%AAI) and 11 orthologs were found in *H. pylori* Shi470 (average 46.6% AAI) (Supplementary Table S6). This suggests that the three organisms may have specific methylation patterns in common and may thus share similar gene regulation, host interaction, pathogenicity or cellular defense systems. Furthermore, we could find 15 putative RNA MTases, all of which were also found in *H. hepaticus* (average 68.6% AAI), while 13 were seen in *H. pylori* Shi470 (average 47.5% AAI) (Supplementary Table S7).

**FIGURE 4:**
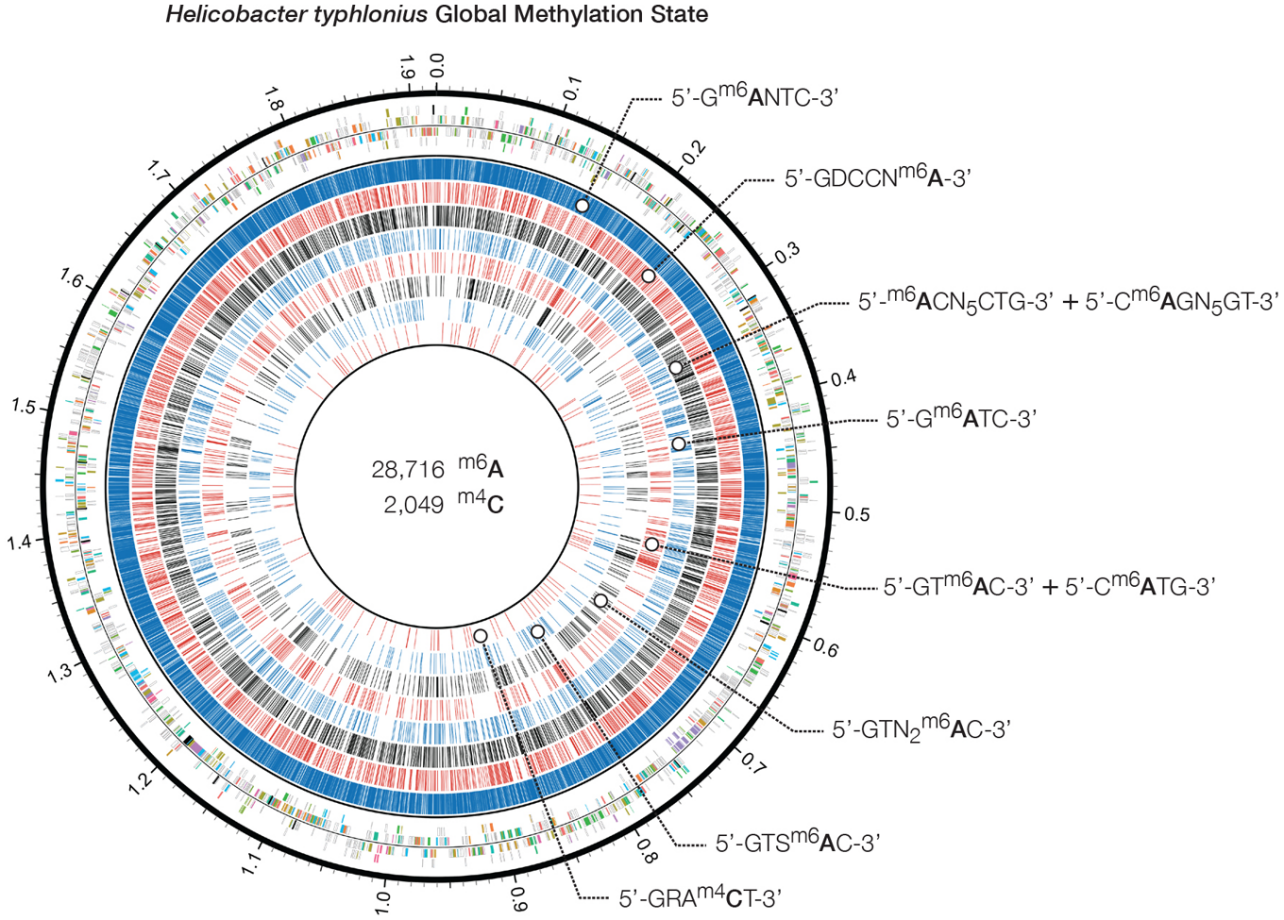
***Heliobacter typhlonius* global methylation state.** Circos plot showing in the outermost ring the protein-encoding genes colored by associated subsystem. The following rings depict methylated adenines and cytosines that are associated with specific motifs. From outside to inside: 5′-G^m6^ANTC-3′ (blue), 5′-GDCCN^m6^A-3′ (red), 5′-GRA^m4^**C**T-3′ (black), 5′-^m6^ACN_5_CTG-3′ and its partner-motif C^m6^AGN_5_GT-3′ (blue), 5′-G^m6^ATC-3′ (red), 5′-GT^m6^AC-3′ and its partner-motif 5′-C^m6^ATG-3′ (black), 5′-GTN_2_^m6^AC-3′ (blue) and 5′-GTS^m6^AC-3′ (red).

Genome-wide analysis of polymerase kinetics during SMRT sequencing enabled the detection of methylated adenine and cytosine bases. The DNA did not receive Tet1 oxidation treatment prior to SMRT sequencing since this requires further fragmentation of the sequencing library, which in turn is not suited for completing the genome of *H. typhlonius*. Without Tet1 treatment only N6-methyladenine (6mA) and 4-methylcytosine (4mC) signals could be reliably detected (Clark et al., 2013). Adenine bases showed a very distinct modification signal that corresponded well with the overall coverage depth on each strand (Supplementary Figures S5 and S6). We found 28,716 6mAs and 2,049 4mCs base modifications that were distributed across the genome. In total 27,399 methylated adenines (95.4%) and 1,977 methylated cytosines (73.7%) were associated with 8 putative MTase recognition motifs (**Figure 4**).

Krebes et al. (2014) performed SMRT sequencing to conduct a comprehensive analysis on two *H. pylori* strains: 26695 and J99-R3. They demonstrated both *pylori* genomes are highly methylated, containing a large number of methyltransferases and restriction–modification systems (Krebes et al., 2014). In contrast, no methylation data is available for *H. hepaticus*, and only two complete R-M systems have been described (Suerbaum et al., 2003). Three out of 8 motifs could be found in both *H. typhlonius* and *H. pylori* strains 26695 and J99-R3: 5′-G^m6^ANTC-3′, 5′-G^m6^ATC-3′ and 5′-GT^m6^AC-3′ with its partner-motif (reverse-complement) C^m6^ATG. One motif was found in *H. typhlonius* and in *pylori* strain J99-R3 only: 5′-GTS^m6^AC-3′. The three remaining motifs were found exclusively in *H. typhlonius*: 5′-GDCCN^m6^A-3′, 5′-^m6^ACN_5_CTG-3′ and its partner-motif C^m6^AGN_5_GT and GTNN^m6^AC. Nearly all of the target sequences were completely methylated (>98%) and resided predominantly in the coding regions of the genome (**Table 4**). Sequence context analysis did not reveal any motifs associated with cytosine methylation.

**Table 4:** Base modifications and motifs: adenine and cytosine motif statistics.

Motif^1^	# Motifs in Genome	# Motifs Detected	% Motifs Detected	% Intergenic	Mean Coverage	Presence in *H. pylori*
G**A**NTC	20,546	20,492	99.7 %	9.3%	237.1	J99-R3, 26695
GDCCN**A**	2,110	2,073	98.2%	3.6%	236.1	
GRA**C**T	2,682	1,977	73.7%	5.6%	233.4	
**A**CN_5_CTG–C**A**GN_5_GT ^∗^	1,980	1,965	99.2%	3.6%	234.7	
G**A**TC	1,166	1,152	98.8%	5.7%	237.1	J99-R3, 26695
GT**A**C-C**A**TG **^∗^**	1,068	1,025	96.0%	6.9%	241.1	J99-R3, 26695**^∗∗^**
GTNN**A**C	512	476	93.0%	3.8%	233.1	
GTS**A**C	222	216	93.7%	7.9%	236.4	J99-R3

*Motifs with a modification quality value >100 are considered.*

*^1^Methylated adenines are typed in bold.*

*^∗^Partner-motifs (motif + its reverse-complement).*

*^∗∗^Motif GT**A**C not reported for strain 26695, the reverse complement, C**A**TG, is found (Krebes et al., 2014).*

## Conclusion

In this study, the complete genome sequence of *H. typhlonius* MIT 97-6810 enabled us to identify many pathogenic features (including a set of 19 possibly pathogenic proteins), the presence of CDTs, a putative PAI (containing components of a T4SS together with a cag protein) and multiple virulence factors. These findings suggest that *H. typhlonius* has the potential to act as a pathobiont.

Furthermore, we described the global methylation state of the genome. We found many methylated sites and discovered a diverse plethora of R-M systems. Methylation patterns differ among closely related species, nonetheless specific recognition motifs are conserved. Together with the genome, the methylome will provide an essential resource for forthcoming studies investigating gene regulation, host interaction, pathogenicity and cellular defense. Follow-up studies are necessary to investigate the pathophysiologic effects of *H. typhlonius* and to evaluate the true nature of its pathogenic capabilities. In turn, these findings can contribute to unraveling the role of *Helicobacter* in enteric disease.

## Materials and Methods

### Genomic DNA Preparation

The *H. typhlonius* strain MIT 97-6810 has been isolated from the cecal and fecal content of *Il10^−^/^−^* knockout mice with IBD by Fox et al. (1999). *H. typhlonius* was obtained from the Culture Collection, University of Gothenburg, Sweden (CCUG 48335T) and was grown micro-aerobically on Biomerieux chocolate agar + PolyViteX (PVX) plates (Mediaproducts, Groningen, The Netherlands) for 2–3 days at 37°C (Franklin et al., 2001). Genomic DNA was extracted using the MOBIO Ultraclean Fecal kit (Sanbio, Uden, The Netherlands) according to the manufacturer’s instructions, combined with phenol–chloroform extraction and RNase A treatment.

### Sequencing

Genomic DNA was fragmented with G-tubes (Covaris), end-repaired and SMRTbell DNA template libraries (insert size of ∼20 Kb) were prepared according to the manufacturer’s specification. SMRT sequencing (3 SMRT cells) was performed on the Pacific Biosciences RSII sequencer according to standard protocols (MagBead Standard Seq v2 loading, 1 × 180 min movie) using the P4-C2 chemistry.

### *De Novo* Genome Assembly

Continuous long reads were attained from three SMRT sequencing runs. Reads longer than 500 bp with a quality value over 0.75 were merged together into a single dataset. Next, the hierarchical genome-assembly process (HGAP) pipeline (Chin et al., 2013) was used to correct for random errors in the long seed reads (seed length threshold 6 Kb) by aligning shorter reads from the same library against them. The resulting corrected, preassembled reads were used for *de novo* assembly using Celera Assembler 8.1 (Myers et al., 2000). Celera Assembler employs an overlap-layout-consensus (OLC) strategy that is well suited for the use of long, corrected PacBio reads. Since SMRT sequencing features very little variations of the quality throughout the reads (Koren et al., 2012), no quality values were used during the assembly. Default parameters were employed while using the BOGART unitigger and setting the *merSize* to 14 (configuration settings are provided in Supplementary File S1). To validate the quality of the assembly and determine the final genome sequence, the Quiver consensus algorithm (Chin et al., 2013) was used. Quiver takes advantage of all information from the raw pulse and base-calls that are generated during the SMRT sequencing to infer the most accurate consensus sequence (Chin et al., 2013). Finally, the ends of the assembled sequence were trimmed to have the genome circularized.

### Annotations

The location of the origin of replication site (*oriC*) was predicted using the Ori-Finder web service (Gao and Zhang, 2008). Ori-Finder was configured to search for *Helicobacter* specific *DnaA* boxes while allowing for two unmatched sites. In addition, the DoriC database (Gao et al., 2013) holding prokaryote *oriC* data was used to select the most likely candidate *oriC* amongst the Ori-Finder results. Annotation of the assembled genome was performed using RAST prokaryotic genome annotation service (Aziz et al., 2008). Additional annotation was carried out using several web services offered by the Center for Genomic Epidemiology. ResFinder 2.1 (Zankari et al., 2012), PathogenFinder 1.1 (Cosentino et al., 2013) and VirulenceFinder 1.2 (Joensen et al., 2014) were used for the prediction of acquired antimicrobial resistance genes, potential pathogenic features and virulence genes respectively. PHAST (PHAge Search Tool) (Zhou et al., 2011) was used to detect and annotate prophage sequences in the assembled genome. CRISPRs were identified using the CRISPRFinder web tool (Grissa et al., 2007). Genomic repeats and other structural variations were identified using NUCmer (Kurtz et al., 2004) and filtered according to length threshold of 500 bp and 95% copy identity. Tandem repeats were identified separately using Tandem Repeat Finder online service (Benson, 1999).

### Comparative Genome Analysis

The final genome sequence of *H. typhlonius* was compared to the genome sequences of two other *Helicobacter* species: murine *H. hepaticus* ATCC 51449 (Suerbaum et al., 2003) and human *H. pylori* 26695 (Krebes et al., 2014). RAST/The SEED was used to infer the conservation of annotated genes and pathways. BLAST (Altschul et al., 1990) searches using default parameters were performed to identify regions of interest.

### Base Modification Analysis

The DNA did not receive Tet1 oxidation treatment prior to SMRT sequencing, meaning only N6-methyladenine (6mA) and 4-methylcytosine (4mC) signals could be reliably detected (Clark et al., 2013). All reads were aligned to the assembled genome. Kinetic signals detected during SMRT sequencing were processed for all genomic positions using a previously described protocol (Flusberg et al., 2010; Clark et al., 2012). The Pacific Biosciences SMRT Portal analysis platform 2.3.0 was used to identify modified bases and associated motifs. The DNA base modification analysis uses an *in silico* kinetic model and a *t*-test based scoring system to detect modified bases. In order to accurately identify methylated bases, a threshold of 100 for log-transformed *P*-value was used. The threshold was optimized according to the distribution of *P*-values for different bases, minimizing the false positive rate. Additional data analysis was performed in R (R Core Team, 2015).

## Author Contributions

JF and SA performed the analyses. SA and ER-M designed the study. CD, AS, and RV performed library preparation and SMRT sequencing. SA, ER-M, G-JvO and JdD coordinated the study. JF drafted the manuscript that was subsequently revised by all co-authors.

## Data Availability

The whole-genome shotgun SMRT sequencing reads of *H. typhlonius* MIT 97-6810 are deposited at the European Nucleotide Archive (ENA) under study ID PRJEB10402 (http://www.ebi.ac.uk/ena/data/view/PRJEB10402). The complete genome sequence and annotation can be retrieved using chromosome accession number LN907858 (http://www.ebi.ac.uk/ena/data/view/LN907858). RAST annotation data is accessible through The Seed Viewer, filed under Genome ID 76936.6. RAST guest account can be used to access all the files that were generated during the annotation and comparative genomics (username: guest; password: guest).

## Conflict of Interest Statement

The authors declare that the research was conducted in the absence of any commercial or financial relationships that could be construed as a potential conflict of interest.
