# Supplementary material for: The Complete Genome Sequence of the Murine Pathobiont Helicobacter typhlonius
Source: Front Microbiol. 2016 Jan 8;6:1549. doi: 10.3389/fmicb.2015.01549 (PMC4705304; doi:10.3389/fmicb.2015.01549)
Supplement: Supplementary file 1 [file Data_Sheet_1.PDF]

Supplementary Material

The complete genome sequence of the murine pathobiont *Helicobacter typhlonius*

Jeroen Frank, Celia Dingemanse-van der Spek, Arnoud M. Schmitz, Rolf H.A.M. Vossen, Gert-Jan B van Ommen, Johan T. den Dunnen, Els C. Robanus-Maandag, Seyed Yahya Anvar*

*** Correspondence:** Seyed Yahya Anvar: s.y.anvar@lumc.nl

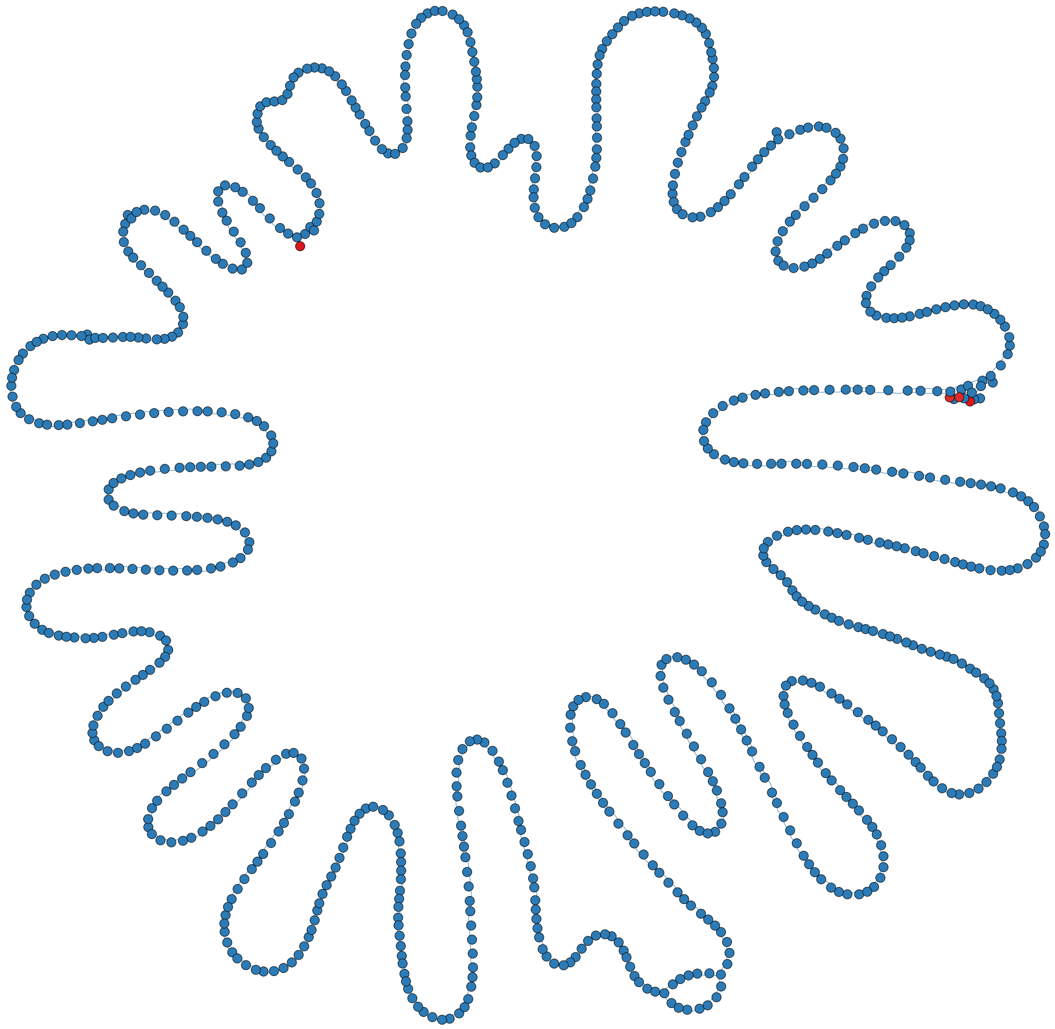

Supplementary Figure 1: Assembly graph. Graph illustrating the overlaps (edges) between the assembled unitigs (vertices). The size and color of the vertices represent unitig size and coverage (blue: > 200x, red: < 20x) respectively.

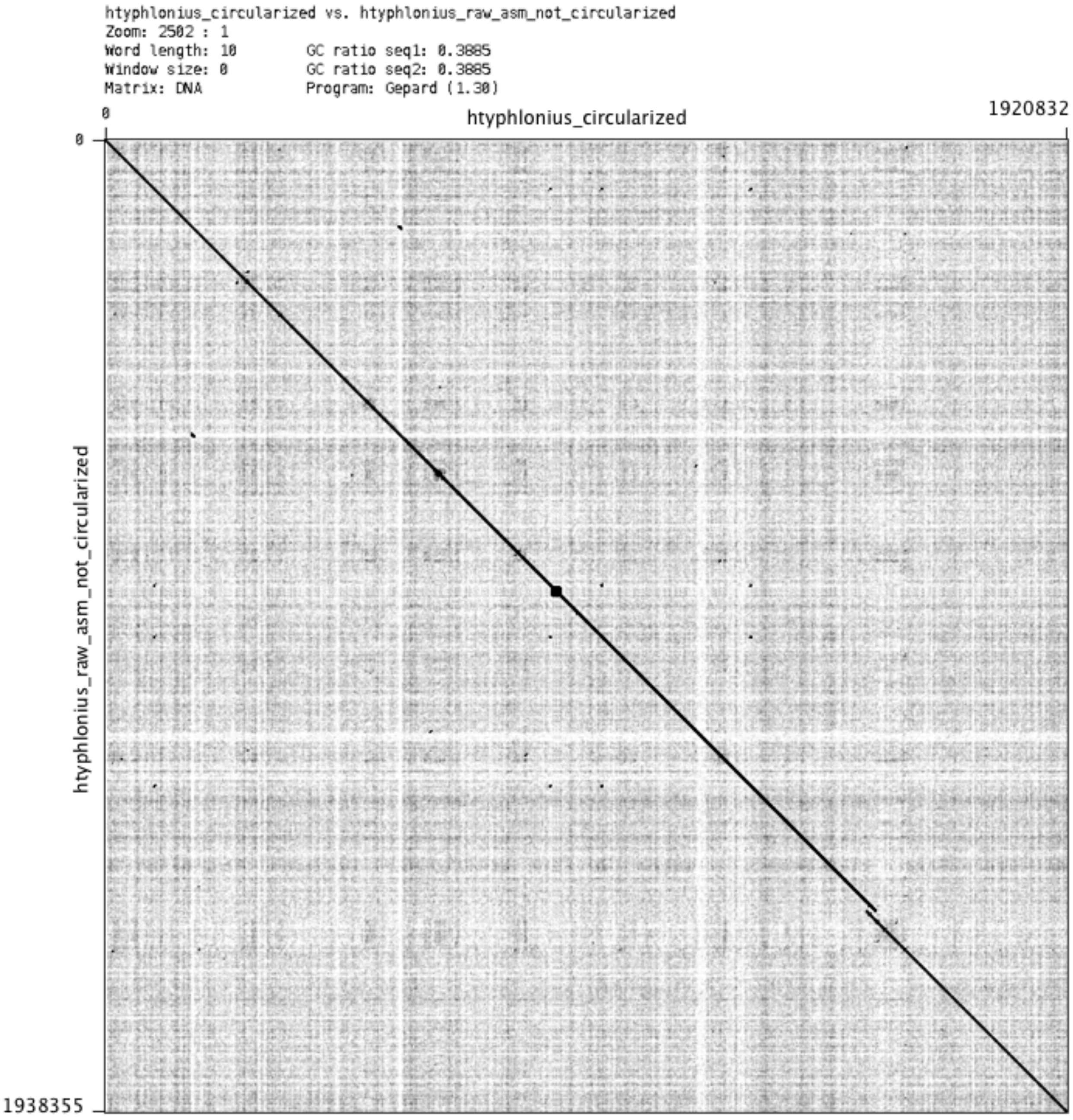

Supplementary Figure 2: Proof of genome completion. Dot plot comparing the initial assembled sequence (y-axis) with the final circularized genome (x-axis). The region showing overlap at the ends of the initial assembled sequence was trimmed to yield the final circularized genome.

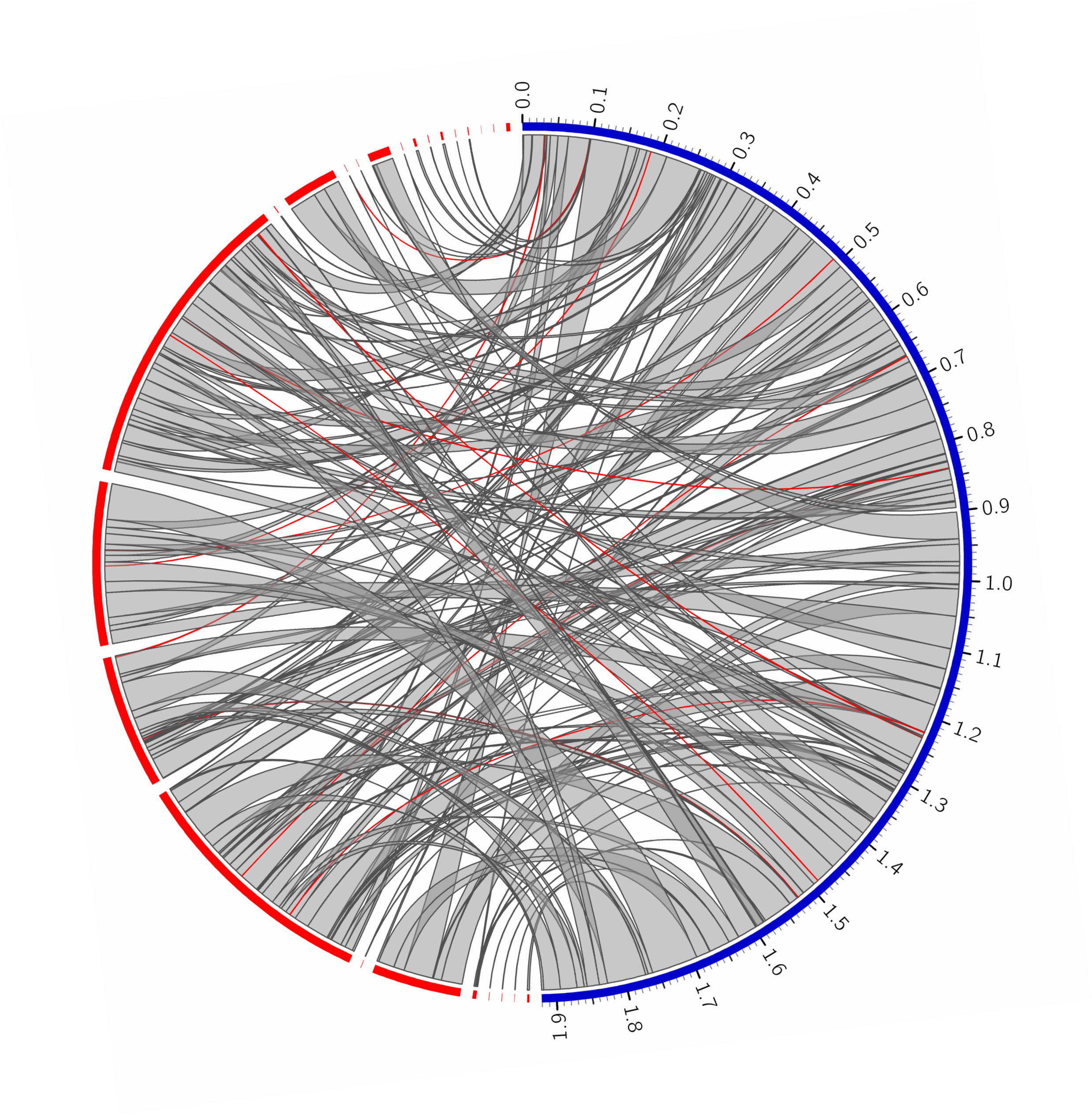

Supplementary Figure 3: Concordance between PacBio assembly and Illumina MiSeq assembly. Circos plot showing all DNA alignments (grey: > 99% identity, red: <99% identity) between the single contig PacBio assembly (blue) and 25 scaffolds assembled from MiSeq reads by Fox *et al.* (red, 127 contigs where scaffolded into 25 scaffolds). GenBank entry: ASM76576v1.

PEG ID	Name	Subsystem	Function
peg.1713	VirB2		Major pilus subunit of type IV secretion complex
peg.1714	VirB4		ATPase provides energy for both assembly of type IV secretion complex and secretion of T-DNA complex
peg.1715	VirB5		Minor pilin of type IV secretion complex
peg.1716	VirB6		Inner membrane protein of type IV secretion of T-DNA complex
peg.1718	VirB8		Inner membrane protein forms channel for type IV secretion of T-DNA complex
peg.1719	VirB9		Outer membrane and periplasm component of type IV secretion of T-DNA complex, has secretin-like domain
peg.1720	VirB10		Inner membrane protein of type IV secretion of T-DNA complex, TonB-like
peg.1721	VirB11		ATPase required for both assembly of type IV secretion complex and secretion of T-DNA complex
peg.1724	VirD4		Coupling protein, ATPase required for T-DNA transfer
peg.1726			Cag pathogenicity island protein
peg.1708			Virulence-associated protein 2
peg.1749			Virulence-associated protein 2
peg.1750			Virulence-associated protein 2
peg.64	CdtA	Cytolethal distending toxin of Campylobacter jejuni:Cdts	Cytolethal distending toxin subunit A
peg.65	CdtB	Cytolethal distending toxin of Campylobacter jejuni; Cdts	Cytolethal distending toxin subunit B
peg.66	CdtC	Cytolethal distending toxin of Campylobacter jejuni; Cdts	Cytolethal distending toxin subunit C

Supplementary Table 1. Overview of pathogenic features.
List of pathogenic features discussed in the main text. All listed PEGs are located in a putative pathogenicity island (genomic location 1,532,276 – 1,597,776 bp), with the exception of Cytolethal distending toxins (CDTs).

PEG ID	Description	*H. hepaticus protein ID*	Description	%AAI
peg.1431	Putative 2-acylglycerophosphoethanolamine acyltransferase	AAP76903	Conserved hypothetical protein	98.80%
peg.395	L-lactate permease	AAP77246	L-lactate permease	100.00%
peg.372	Putative two-domain glycosyltransferase	AAP77370	Conserved hypothetical protein	98.01%
peg.393	Predicted L-lactate dehydrogenase, Iron-sulfur cluster-binding subunit YkgF	AAP77248	Conserved hypothetical protein containing a ferredoxin-like domain	96.45%
peg.1468	Multi antimicrobial extrusion protein	AAP76764	Conserved hypothetical membrane protein	96.72%
peg.1889	Putative transmembrane transport protein	AAP77291	Conserved hypothetical protein	96.37%
peg.1866	FIG00711941 hypothetical protein	AAP77620	Conserved hypothetical protein	97.03%
peg.618	Tryptophanyl-tRNA synthetase	AAP77155	Tryptophanyl-tRNA synthetase	98.49%
peg.379	tRNA dimethylallyltransferase (EC 2.5.1.75)tRNA processing	AAP77507	tRNA delta(2)-isopentenylpyrophosphate transferase	98.68%
peg.369	ABC transporter ATP binding protein	AAP77373	ABC transporter	99.19%
peg.394	Predicted L-lactate dehydrogenase, Fe-S oxidoreductase subunit YkgE Lactate utilization	AAP77247	Conserved hypothetical protein	100.00%
peg.1941	FIG00712993: hypothetical protein	AAP77307	Conserved hypothetical protein	99.57%
peg.41	FIG00710446: hypothetical protein	AAP77654	Conserved hypothetical protein	100.00%
peg.1373	Transcriptional regulator	AAP78057	Conserved hypothetical protein	97.66%
peg.80	Hypothetical protein	AAP77693	Hypothetical protein	100.00%
peg.1606	FIG00712120: hypothetical protein	AAP77341	Conserved hypothetical protein	96.38%
peg.409	FIG00711357: hypothetical protein	AAP78098	Conserved hypothetical protein	99.07%

Supplementary Table 2. PathogenFinder results.
PEGs linked to pathogenic families in *H. Hepaticus* ATCC 51449.

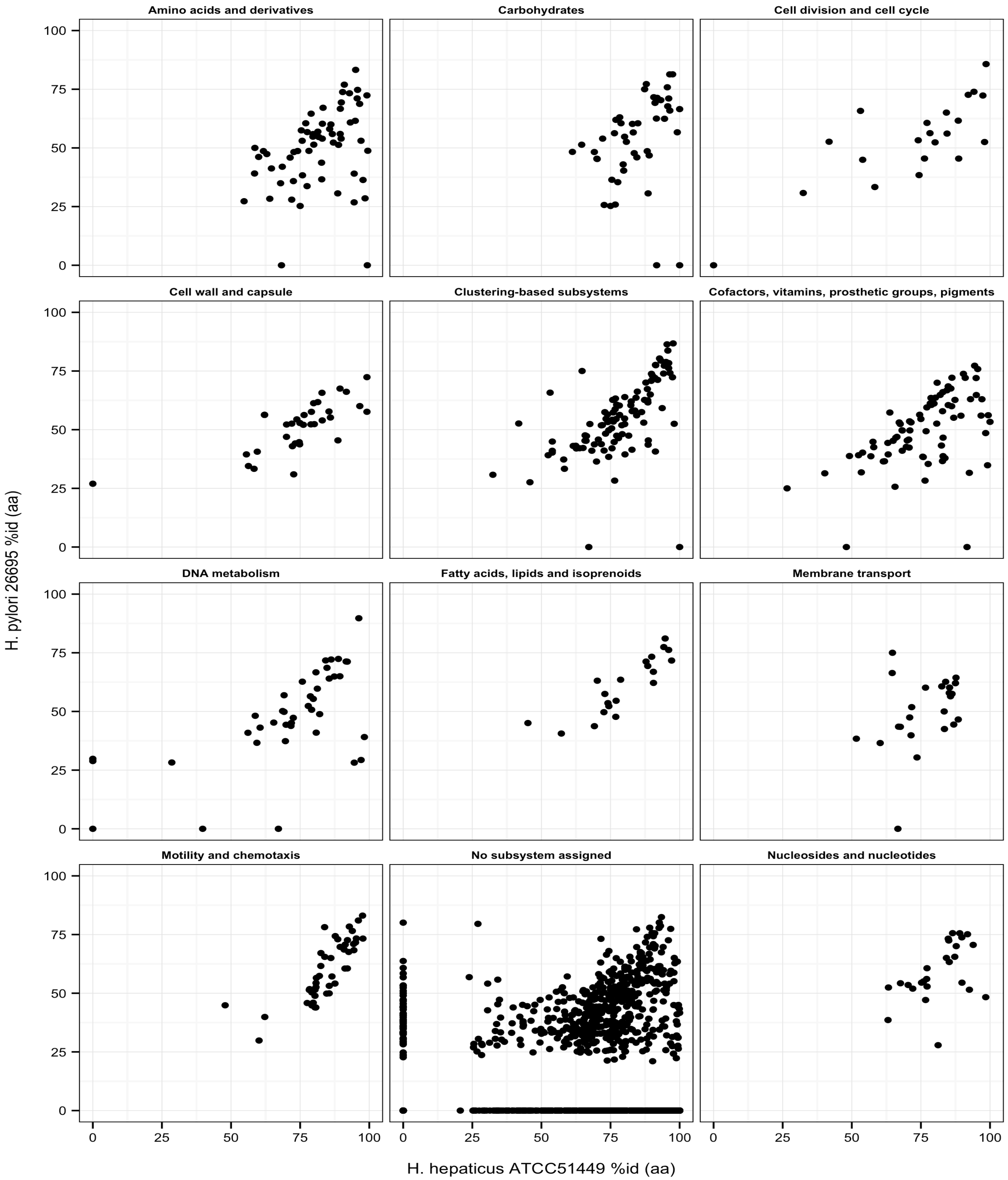

Supplementary Figure 4: Conservation of major subsystems in *H. hepaticus* and *H. pylori.* Scatterplots showing the amino acid identity (AAI) of PEGs assigned to major subsystems in *H. hepaticus* ATCC51449 (x-axis) and *H. pylori* 26695 (y-axis).

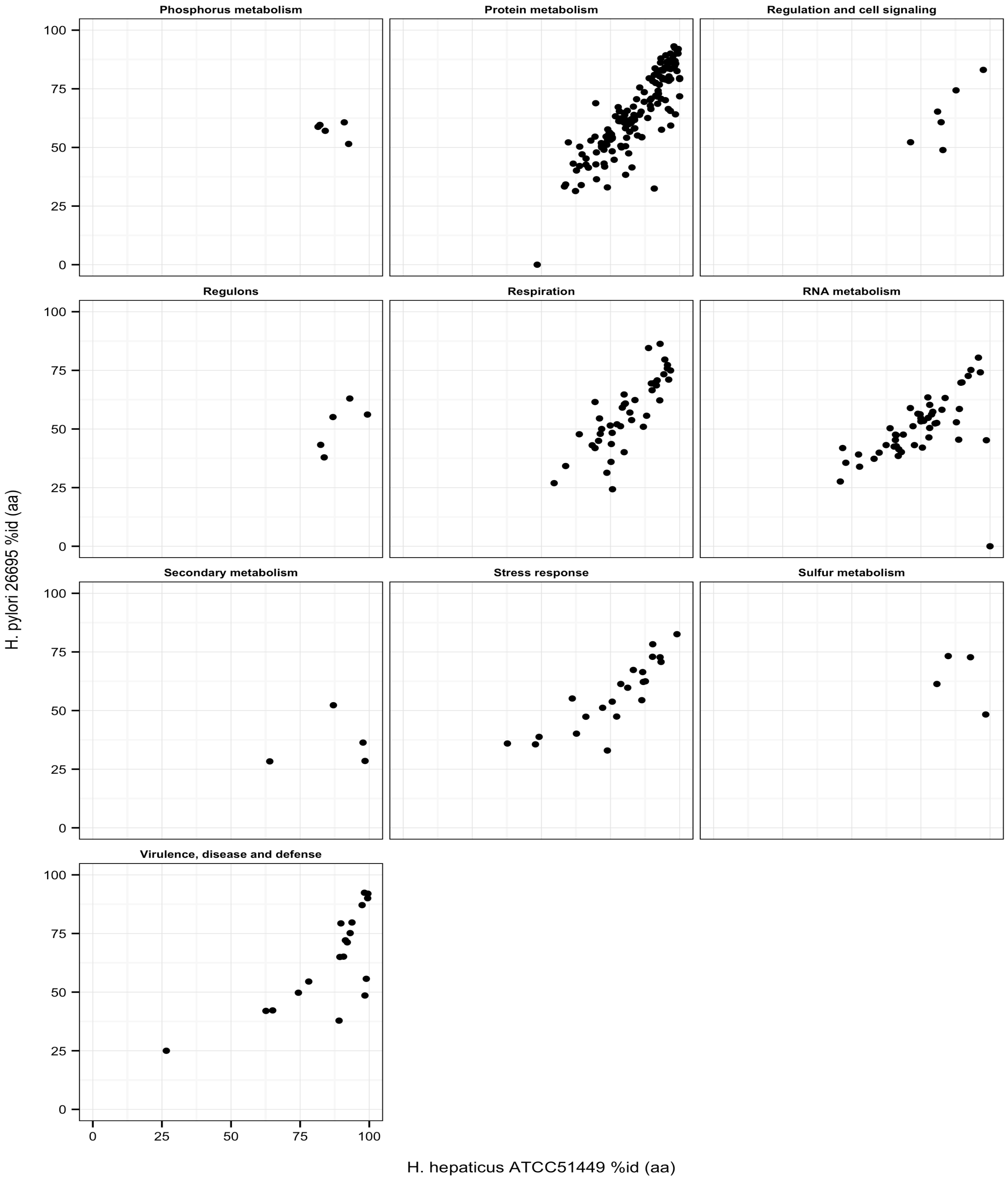

Supplementary Figure 4 (continued): Conservation of major subsystems in *H. hepaticus* and *H. pylori.*

PEG ID	Subsystem	Function
peg.26	DNA phosphorothioation	3'-phosphoadenosine 5'-phosphosulfate sulfurtransferase DndC
peg.27	DNA phosphorothioation	DNA sulfur modification protein DndD
peg.52		Methyltransferase
peg.56		Capsular polysaccharide biosynthesis protein
peg.57		Capsular polysaccharide biosynthesis protein
peg.75	Restriction-Modification System	Putative DNA-binding protein in cluster Type I restriction-modification system
peg.109	Folate biosynthesis cluster; Purine conversions	Hypoxanthine-guanine phosphoribosyltransferase (EC 2.4.2.8)
peg.110	Folate biosynthesis cluster; Purine conversions	Hypoxanthine-guanine phosphoribosyltransferase (EC 2.4.2.8)
peg.130		Glycosyltransferase
peg.131		Glycosyltransferase
peg.146		Huntingtin interacting protein E-like protein
peg.219		Phage (Mu-like) virion morphogenesis protein
peg.220	Phage capsid proteins	Phage capsid and scaffold
peg.274		Voltage-gated sodium channel subunit
peg.286		Rhodanese-like domain protein
peg.345		Putative type IIS restriction/modification enzyme
peg.390		Putative type IIS restriction /modification enzyme, N-terminal half
peg.513		RfbJ protein
peg.592		Cell filamentation protein fic
peg.634	Flagellar motility	Signal transduction protein CetB, mediates an energy taxis response
peg.641		4-hydroxybutyrate coenzyme A transferase
peg.687		Major facilitator family transporter, putative
peg.689		D amino acid oxidase (DAO) family (EC 1.4.3.3)
peg.691		Glycosyl transferase, family 2
peg.692		Glycosyl transferase, group 2 family protein (EC:2.4.1.-)
peg.720		Arsenical resistance operon repressor
peg.721		Zinc finger domain-containing protein
peg.735		Outer membrane efflux protein
peg.791		HlpA protein
peg.801		glycosyl transferase, group 1 family protein

Supplementary Table 3. List of annotated PEGs exclusive to *H. typhlonius* compared to *H. hepaticus* and *H. pylori.* Pegs exclusively present in *H. typhlonius* in the sense that there are no orthologous sequences found in both *H. hepaticus and H. pylori.*

PEG ID	Subsystem	Function
peg.1132		BtrN protein
peg.1170		Sugar transferase
peg.1171		HlpA protein
peg.1202		Generic methyltransferase
peg.1289		Capsular polysaccharide biosynthesis protein-like protein
peg.1297		Pyridoxal phosphate-dependent deaminase, putative
peg.1313	Flagellar motility	Signal transduction protein CetB, mediates an energy taxis response
peg.1359		LemA protein
peg.1364		Type II restriction enzyme
peg.1375	Type I Restriction-Modification	Type I restriction-modification system, specificity subunit S (EC 3.1.21.3)
peg.1429		Putative 2-acylglycerophosphoethanolamine acyltransferase acyl-acyl carrier protein synthetase (EC 6.2.1.20)
peg.1456	2-phosphoglycolate salvage; glyoxylate interconversion	Phosphoglycolate phosphatase (EC 3.1.3.18)
peg.1464		IroE protein
peg.1470		GtrA-like protein
peg.1472		Putative inner membrane protein
peg.1475		Bactoprenol glucosyl transferase (EC 2.4.1.-)
peg.1478		Glycosyltransferase
peg.1541		Putative type IIS restriction /modification enzyme, N-terminal half
peg.1627		WbuO
peg.1674		Methyltransferase FkbM
peg.1702		Putative methyltransferase
peg.1712		Site-specific recombinase, resolvase family
peg.1713		Major pilus subunit of type IV secretion complex, VirB2
peg.1715		Minor pilin of type IV secretion complex, VirB5
peg.1716		Inner membrane protein of type IV secretion of T-DNA complex, VirB6
peg.1731		Micrococcal nuclease (thermonuclease) homologs
peg.1733		Helicase, Snf2 family

Supplementary Table 3 (continued). List of annotated PEGs exclusive to *H. typhlonius* compared to *H. hepaticus* and *H. pylori.*

PEG ID	Subsystem	Function
peg.1737		Probable ATPase
peg.1738		IncQ plasmid conjugative transfer DNA nicking endonuclease TraR (pTi VirD2 homolog)
peg.1739		Ribbon-helix-helix protein, copG family domain protein
peg.1743	Macromolecular synthesis operon	DNA primase (EC 2.7.7.-)
peg.1746		Putative two-component system sensor histidine kinase, putative heat shock protein
peg.1749		Virulence-associated protein 2
peg.1750		Virulence-associated protein 2
peg.1752	Aspartate and Asparagine Biosynthesis	Asparagine synthetase [glutamine-hydrolyzing] (EC 6.3.5.4)
peg.1754		Radical SAM domain protein
peg.1759		Cationic amino acid transporter
peg.1760		Putative sugar transferase
peg.1761		Putative sugar transferase
peg.1765	CRISPRs	CRISPR-associated protein Cas1
peg.1766	CRISPRs	CRISPR-associated protein Cas2
peg.1769		MFS transporter
peg.1774		Predicted phosphatase
peg.1831	DNA ligases	DNA ligase (ATP) (EC 6.5.1.1)
peg.1929		Putative transcriptional regulator
peg.1938	Ton and Tol transport systems	Putative Ton-B dependent hemine receptor
peg.1951		Endonuclease I precursor (EC 3.1.21.1)
peg.1972	DNA repair, bacterial	DNA recombination protein RmuC
peg.2024		Transcriptional regulator, TetR family
peg.2026	Aspartate and Asparagine Biosynthesis	Glutamate synthase [NADPH] large chain (EC1.4.1.13)
peg.2048		BtrN protein
peg.2110		5-nitroimidazole antibiotic resistance protein

Supplementary Table 3 (continued). List of annotated PEGs exclusive to *H. typhlonius* compared to *H. hepaticus* and *H. pylori.*

PEG ID	Subsystem	Function
peg.3		Modification methylase
peg.12		Transcriptional regulator, HxlR family
peg.18	FMN and FAD metabolism in plants	Multi antimicrobial extrusion protein (Na+/drug antiporter), MATE family of MDR efflux pumps
peg.57		Capsular polysaccharide biosynthesis protein
peg.75	Restriction-Modification System	Putative DNA-binding protein in cluster with Type I restriction-modification system
peg.108		Putative ABC transporter ATP binding protein
peg.109	Folate biosynthesis cluster; Purine conversions	Hypoxanthine-guanine phosphoribosyltransferase (EC 2.4.2.8)
peg.110	Folate biosynthesis cluster; Purine conversions	Hypoxanthine-guanine phosphoribosyltransferase (EC 2.4.2.8)
peg.123		Methyl-accepting chemotaxis signal transduction protein
peg.131		Glycosyltransferase
peg.196		Putative processing protease
peg.320		N6 adenine-specific DNA methyltransferase, N12 class
peg.345		Putative type IIS restriction/modification enzyme
peg.477		Carbonic anhydrase (EC 4.2.1.1)
peg.544	ABC transporter dipeptide (TC 3.A.1.5.2)	Dipeptide transport system permease protein DppC (TC 3.A.1.5.2)
peg.603		Type IV secretion/competence protein (VirB10)
peg.628		Outer membrane protein assembly factor YaeT precursor
peg.641		4-hydroxybutyrate coenzyme A transferase
peg.713	Type I Restriction-Modification	Type I restriction-modification system, specificity subunit S (EC 3.1.21.3)
peg.773	Alanine biosynthesis	Cysteine desulfurase (EC 2.8.1.7)
peg.1164		HIT family protein
peg.1247		Hydroxymethylpyrimidine phosphate kinase ThiD (EC 2.7.4.7)
peg.1297		pyridoxal phosphate-dependent deaminase, putative
peg.1358		Heat shock protein HtpX / FIG017973: domain of unknown function
peg.1359		LemA protein
peg.1366		Aldo-keto reductase
peg.1376		Type II restriction-modification enzyme
peg.1475		Bactoprenol glucosyl transferase (EC 2.4.1.-)
peg.1515		Adenine specific DNA methyltransferase
peg.1702		Putative methyltransferase

Supplementary Table 4. Annotated PEGs unique to *H. typhlonius* compared to all other Helicobacters in The SEED database.

PEG ID	Subsystem	Function
peg.1720		Inner membrane protein of type IV secretion of T-DNA complex, TonB-like, VirB10
peg.1743	Macromolecular synthesis operon	DNA primase (EC 2.7.7.-)
peg.1745	DNA repair, bacterial	DNA-cytosine methyltransferase (EC 2.1.1.37)
peg.1746		Putative two-component system sensor histidine kinase, putative heat shock protein
peg.1752	Glutamate, Aspartate and Asparagine Biosynthesis	Asparagine synthetase [glutamine-hydrolyzing] (EC 6.3.5.4)
peg.1755		Glycerol-3-phosphate cytidylyltransferase (EC 2.7.7.39)
peg.2044		Amino acid ABC transporter, periplasmic amino acid-binding portion
peg.2047		Cystine ABC transporter, ATP-binding protein

Supplementary Table 4 (continued). Annotated PEGs unique to *H. typhlonius* compared to all other Helicobacters in The SEED database.

**PEG ID**	**Subsystem**	**Function**	***H. hepaticus* ATCC51449**	***H. pylori* 26695**
peg.74	Restriction-Modification System	Type III restriction-modification system DNA endonuclease res (EC 3.1.21.5)	NA	30.84% (peg.1358)
peg.75	Restriction-Modification System	Putative DNA-binding protein in cluster with Type I restriction-modification system	NA	NA
peg.78	Restriction-Modification System	Type III restriction-modification system methylation subunit (EC 2.1.1.72)	100.00% (peg.1095)	38.74% (peg.1357)
peg.707	Restriction-Modification System	Type I restriction-modification system, DNA-methyltransferase subunit M (EC 2.1.1.72)	NA	29.82% (peg.1390)
peg.708	Restriction-Modification System	Type I restriction-modification system, DNA-methyltransferase subunit M (EC 2.1.1.72)	NA	28.25% (peg.841)
peg.713	Restriction-Modification System	Type I restriction-modification system, specificity subunit S (EC 3.1.21.3)	30.20% (peg.1434)	NA
peg.717	Restriction-Modification System	Type I restriction-modification system, restriction subunit R (EC 3.1.21.3)	NA	28.87% (peg.1389)
peg.916	Restriction-Modification System	Type I restriction-modification system, restriction subunit R (EC 3.1.21.3)	94.58% (peg.1418)	28.18% (peg.837)
peg.921	Restriction-Modification System	Type I restriction-modification system, specificity subunit S (EC 3.1.21.3)	71.83% (peg.1421)	45.10% (peg.781)
peg.923	Restriction-Modification System	Type I restriction-modification system, DNA-methyltransferase subunit M (EC 2.1.1.72)	97.05% (peg.1423)	29.32% (peg.1390)
peg.934	Restriction-Modification System	Type III restriction-modification system methylation subunit (EC 2.1.1.72)	89.16% (peg.1445)	32.26% (peg.1615)
peg.1363	Restriction-Modification System	Type III restriction-modification system methylation subunit (EC 2.1.1.72)	90.36% (peg.1445)	34.38% (peg.1615)
peg.1375	Restriction-Modification System	Type I restriction-modification system, specificity subunit S (EC 3.1.21.3)	NA	NA

Supplementary Table 5. Components of Restriction-Modification systems in *H. typhlonius.*
Listed are components of R-M systems found in *H. typhlonius.* The last two columns denote orthologous genes in *H. hepaticus* and *H. pylori* respectively. The amount of conservation of the PEG is expressed as the percent amino acid identity (AAI).

PEG ID	Subsystem	Function	*H. hepaticus* ATCC51449	*H. pylori* 26695
peg.3		Modification methylase	NA	45.53% (peg.1340)
peg.25	DNA repair	Methylated-DNA--protein-cysteine methyltransferase (EC 2.1.1.63)	98.24% (peg.1037)	39.10% (peg.668)
peg.35		Ulcer associated adenine specific DNA methyltransferase	97.92% (peg.1050)	58.75% (peg.1196)
peg.52		Methyltransferase	NA	NA
peg.78	Restriction-Modification System	Type III restriction-modification system methylation subunit (EC 2.1.1.72)	100.00% (peg.1095)	38.74% (peg.1357)
peg.320		N6 adenine-specific DNA methyltransferase, N12 class	30.37% (peg.798)	54.10% (peg.899)
peg.707	Restriction-Modification System	Type I restriction-modification system, DNA-methyltransferase subunit M (EC 2.1.1.72)	NA	29.82% (peg.1390)
peg.708	Restriction-Modification System	Type I restriction-modification system, DNA-methyltransferase subunit M (EC 2.1.1.72)	NA	28.25% (peg.841)
peg.923	Restriction-Modification System	Type I restriction-modification system, DNA-methyltransferase subunit M (EC 2.1.1.72)	97.05% (peg.1423)	29.32% (peg.1390)
peg.932		Type II DNA modification enzyme (methyltransferase)	NA	79.56% (peg.90)
peg.934	Restriction-Modification System	Type III restriction-modification system methylation subunit (EC 2.1.1.72)	89.16% (peg.1445)	32.26% (peg.1615)
peg.1202		Generic methyltransferase	NA	NA
peg.1363	Restriction-Modification System	Type III restriction-modification system methylation subunit (EC 2.1.1.72)	90.36% (peg.1445)	34.38% (peg.1615)
peg.1372		Ulcer associated adenine specific DNA methyltransferase	96.44% (peg.1050)	58.16% (peg.1196)
peg.1515		Adenine specific DNA methyltransferase	NA	33.51% (peg.1341)

Supplementary Table 6. DNA methyltransferases in *H. typhlonius*.
Listed are components of DNA MTases found in *H. typhlonius*. . The last two columns denote orthologous genes in *H. hepaticus* and *H. pylori* respectively. The amount of conservation of the PEG is expressed as the percent amino acid identity (AAI).

PEG ID	Subsystem	Function	*H. hepaticus* ATCC51449	*H. pylori* 26695
peg.1702		Putative methyltransferase	NA	NA
peg.1745	DNA repair	DNA-cytosine methyltransferase (EC 2.1.1.37)	NA	33.33% (peg.1590)
peg.1869	DNA repair	Methylated-DNA--protein-cysteine methyltransferase (EC 2.1.1.63)	58.75% (peg.1037)	48.15% (peg.668)

Supplementary Table 6 (continued). DNA methyltransferases in *H. typhlonius.*

PEG ID	Subsystem	Function	*H. hepaticus* ATCC51449	*H. pylori* 26695
peg.117	RNA methylation	LSU m3Psi1915 methyltransferase RlmH	63.85% (peg.1237)	50.32% (peg.938)
peg.191	RNA methylation	Ribosomal RNA small subunit methyltransferase E (EC 2.1.1.-)	47.65% (peg.993)	35.59% (peg.370)
peg.262	RNA methylation	tRNA (Guanine37-N1) -methyltransferase (EC 2.1.1.31)	75.00% (peg.936)	53.25% (peg.1135)
peg.361	RNA methylation	tRNA (guanine46-N7-)-methyltransferase (EC 2.1.1.33)	67.93% (peg.785)	40.15% (peg.738)
peg.416	RNA methylation	16S rRNA (guanine(966)-N(2))-methyltransferase (EC 2.1.1.171) rRNA m(2)G966	59.90% (peg.1553)	39.90% (peg.801)
peg.631		RNA binding methyltransferase FtsJ like	63.49% (peg.519)	41.49% (peg.107)
peg.647	RNA methylation	tRNA (cytidine(34)-2'-O)-methyltransferase (EC 2.1.1.207)	80.13% (peg.378)	NA
peg.948	16S rRNA modification within P site of ribosome	rRNA small subunit methyltransferase I	66.08% (peg.1745)	47.37% (peg.545)
peg.949	RNA methylation	23S rRNA (guanosine-2'-O-) -methyltransferase rlmB (EC 2.1.1.-)	78.17% (peg.1746)	50.45% (peg.546)
peg.1694	RNA methylation	rRNA small subunit 7-methylguanosine (m7G) methyltransferase GidB	75.54% (peg.1486)	42.05% (peg.1050)
peg.1784	RNA methylation	tRNA (adenine37-N(6))-methyltransferase TrmN6 (EC 2.1.1.223)	65.31% (peg.837)	42.49% (peg.1491)
peg.1858	RNA methylation	tRNA (uracil(54)-C5)-methyltransferase (EC 2.1.1.35)	55.71% (peg.693)	NA
peg.1878	16S rRNA modification within P site of ribosome	rRNA small subunit methyltransferase H	66.02% (peg.1279)	47.59% (peg.699)

Supplementary Table 7. RNA methyltransferases in *H. typhlonius.*
Listed are RNA MTases found in *H. typhlonius.* The last two columns denote orthologous genes in *H. hepaticus* and *H. pylori* respectively. The amount of conservation of the PEG is expressed as the percent amino acid identity (AAI).

PEG ID	Subsystem	Function	***H. hepaticus* ATCC51449**	***H. pylori* 26695**
peg.2056		Possible RNA methyltransferase aq_898	86.17% (peg.1198)	67.15% (peg.13)
peg.2075	RNA methylation	Ribosomal RNA large subunit methyltransferase N (EC 2.1.1.-)	78.19% (peg.1178)	60.29% (peg.1415)

Supplementary Table 7 (continued). RNA methyltransferases in *H. typhlonius.*

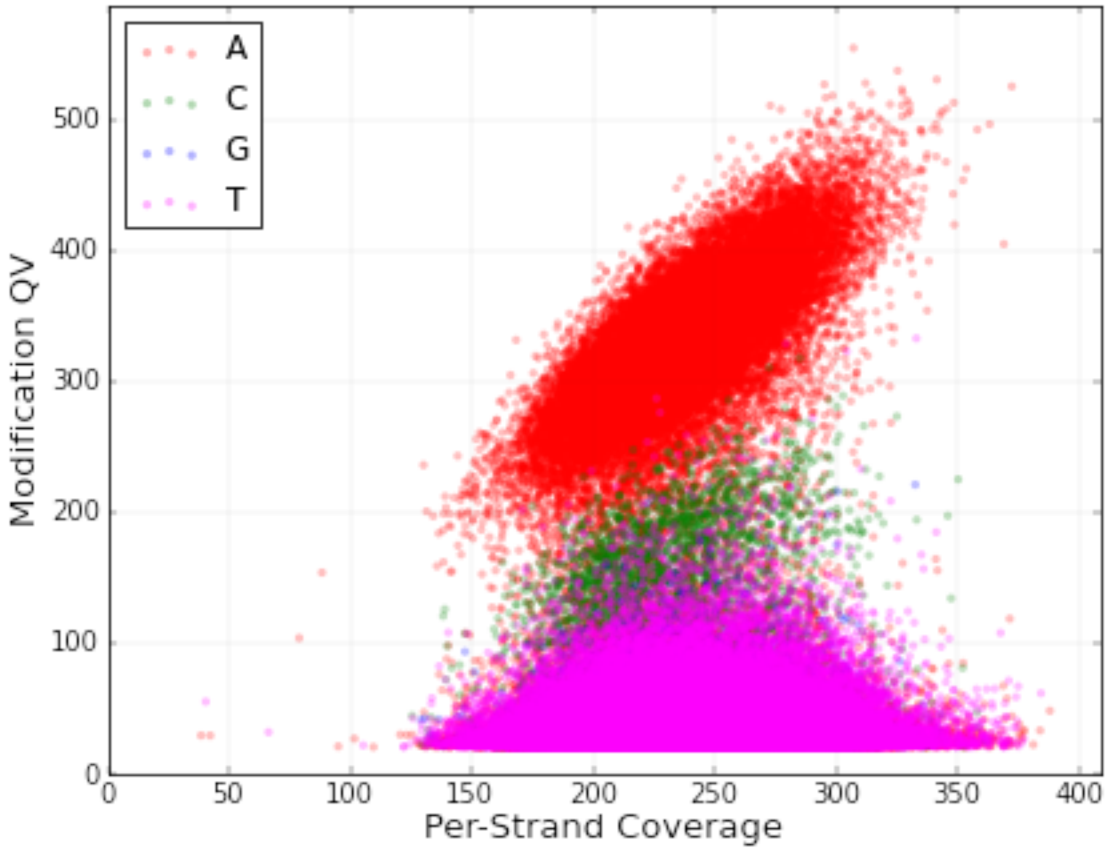

Supplementary Figure 5: Modification quality values compared to coverage per strand.
Average quality values for modification of each base in the *Helicobacter typhlonius* genome are compared to the strand-specific coverage at each genomic position.

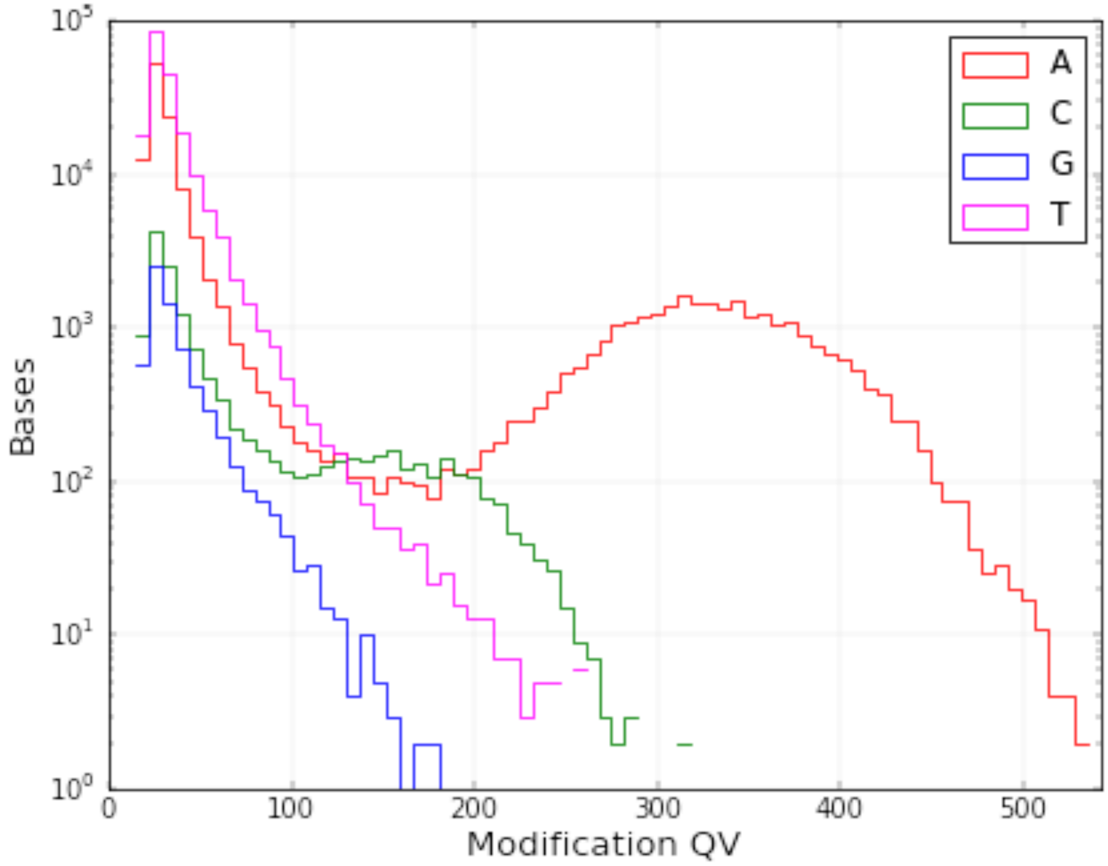

Supplementary Figure 6: Modification quality value distributions per base.
Average quality values for modification of each base in the *Helicobacter typlonius* genome are used to estimate the overall distribution and to determine appropriate threshold for reliable identification of methylated bases.

Supplementary File 1: Celera assembler 8.1 configuration settings.
Configuration settings (“spec file”) used by Celera assembler 8.1 for the *de novo* assembly of corrected, preassembled (HGAP) SMRT sequencing reads. Settings controlling the performance and optimization of the computer cluster have been excluded.

merSize	= 14
overlapper	= ovl
ovlMinLen	= 40
unitigger	= bogart
utgBubblePopping	= 1
doToggle	= 0
toggleNumInstances	= 0
toggleUnitigLength	= 2000
doOverlapBasedTrimming	= 1
doExtendClearRanges	= 2
cgwDemoteRBP	= 0
cgwMergeMissingThreshold	= 0
